# Oral health-related quality of life in Loeys-Dietz syndrome, a rare connective tissue disorder: an observational cohort study

**DOI:** 10.1186/s13023-019-1250-y

**Published:** 2019-12-16

**Authors:** Quynh C. Nguyen, Olivier Duverger, Rashmi Mishra, Gabriela Lopez Mitnik, Priyam Jani, Pamela A. Frischmeyer-Guerrerio, Janice S. Lee

**Affiliations:** 1 Craniofacial Anomalies and Regeneration Section, National Institute of Dental and Craniofacial Research, National Institutes of Health, Bethesda, MD USA; 2 Program Analysis and Reporting Branch, National Institute of Dental and Craniofacial Research, National Institutes of Health, Bethesda, MD USA; 3 Food Allergy Research Unit, National Institute of Allergy and Infectious Diseases, National Institutes of Health, Bethesda, MD USA

**Keywords:** Rare diseases, Rare connective tissue disorders, Loeys-Dietz syndrome (LDS), Oral health-related quality of life (OHRQoL), Oral health impact profile (OHIP-14)

## Abstract

**Background:**

Loeys-Dietz syndrome (LDS) is a rare connective tissue disorder whose oral manifestations and dental phenotypes have not been well-characterized. The aim of this study was to explore the influence of oral manifestations on oral health-related quality of life (OHRQoL) in LDS patients.

**Material and methods:**

LDS subjects were assessed by the craniofacial team at the National Institutes of Health Clinical Center Dental Clinic between June 2015 and January 2018. Oral Health Impact Profile (OHIP-14) questionnaire, oral health self-care behavior questionnaire and a comprehensive dental examination were completed for each subject. OHRQoL was assessed using the OHIP-14 questionnaire with higher scores corresponding to worse OHRQoL. Regression models were used to determine the relationship between each oral manifestation and the OHIP-14 scores using a level of significance of *p* ≤ 0.05.

**Results:**

A total of 33 LDS subjects (51.5% female) aged 3–57 years (19.6 ± 15.1 years) were included in the study. The OHIP-14 scores (*n* = 33) were significantly higher in LDS subjects (6.30 [SD 6.37]) when compared to unaffected family member subjects (1.50 [SD 2.28], *p* < 0.01), and higher than the previously reported scores of the general U.S. population (2.81 [SD 0.12]). Regarding oral health self-care behavior (*n* = 32), the majority of LDS subjects reported receiving regular dental care (81%) and maintaining good-to-excellent daily oral hygiene (75%). Using a crude regression model, worse OHRQoL was found to be associated with dental hypersensitivity (β = 5.24; *p* < 0.05), temporomandibular joints (TMJ) abnormalities (β = 5.92; *p* < 0.01), self-reported poor-to-fair oral health status (β = 6.77; *p* < 0.01), and cumulation of four or more oral manifestations (β = 7.23; *p* < 0.001). Finally, using a parsimonious model, self-reported poor-to-fair oral health status (β = 5.87; *p* < 0.01) and TMJ abnormalities (β = 4.95; *p* < 0.01) remained significant.

**Conclusions:**

The dental hypersensitivity, TMJ abnormalities, self-reported poor-to-fair oral health status and cumulation of four-or-more oral manifestations had significant influence on worse OHRQoL. Specific dental treatment guidelines are necessary to ensure optimal quality of life in patients diagnosed with LDS.

## Background

A rare disease is defined as a condition that affects less than 200,000 Americans. There are currently over 7000 identified rare diseases. Thus, all rare diseases taken together affect approximately 25–30 million Americans, and together affect as many Americans as diabetes [1, 2]. Despite the vast number of Americans living with a rare disease, there is a gap in knowledge on the oral manifestations, appropriate oral and dental treatment and information on oral health-related quality of life (OHRQoL) in these populations [3]. As such, there is a need to understand the importance of OHRQoL in patients afflicted with a rare disease. One of the most common measures of OHRQoL is the Oral Health Impact Profile (OHIP-14) questionnaire which captures functional and psycho-social impairment aggravated by an oral health condition [4]. The validity and reliability of this measure are well-established and have been described and utilized in numerous studies [4–7]. Previous studies have shown that rare connective tissue disorders such as Marfan syndrome, osteogenesis imperfecta (OI), Ehlers–Danlos syndrome and other rare orofacial diseases exhibit oral manifestations and alteration of craniofacial development that appear to negatively impact OHRQoL [8–11].

Loeys-Dietz syndrome (LDS) is a rare connective tissue disorder in which oral manifestations have not been well-characterized and information about the dental phenotype and OHRQoL is lacking [12–15]. LDS arises due to dysregulation of the transforming growth factor-beta (TGF-β) signaling pathway, which disrupts cellular functions such as cell growth, cell differentiation, and cellular homeostasis [16]. The disease is caused by mutations in the *TGFBR1*, *TGFBR2*, *SMAD2*, *SMAD3*, *TGFB2*, or *TGFB3* genes [13, 15]. Although it is a very rare connective tissue disorder with unknown prevalence, mutations in *TGFBR1* and *TGFBR2* appear to be the most common forms [17, 18]. This condition arises predominantly due to de novo pathogenic variants (75%) and is inherited in an autosomal dominant (25%) manner with variable clinical expression. LDS, with features similar to those in Marfan syndrome, is characterized by the clinical triad of aortic root aneurysm and arterial tortuosity, hypertelorism, and bifid uvula or cleft palate [14, 15]. Due to the higher propensity for severe cardiac and vascular problems at an early age compared to Marfan syndrome [19], it is imperative that LDS patients receive early and adequate treatment with involvement of multiple specialists, including dental specialists. Except for the recommendation of subacute bacterial endocarditis prophylaxis for individuals undergoing dental work to prevent secondary complications [13, 15], little is known about the burden of oro-dental anomalies faced by this group, their dental needs, and their quality of life based on their oral manifestations.

The purpose of this study was to (i) define the OHRQoL status of LDS patients and (ii) determine the relationship between OHIP-14 scores and oral manifestations in these patients. We hypothesized that individuals with LDS have more identifiable dental problems and worse OHRQoL compared to the general population. To test these hypotheses, patients with LDS underwent a comprehensive oral examination with dental phenotyping as well as completed a self-reported oral health assessment and the OHIP-14 questionnaire.

## Material and methods

### Study participants

**Fig. 1:**
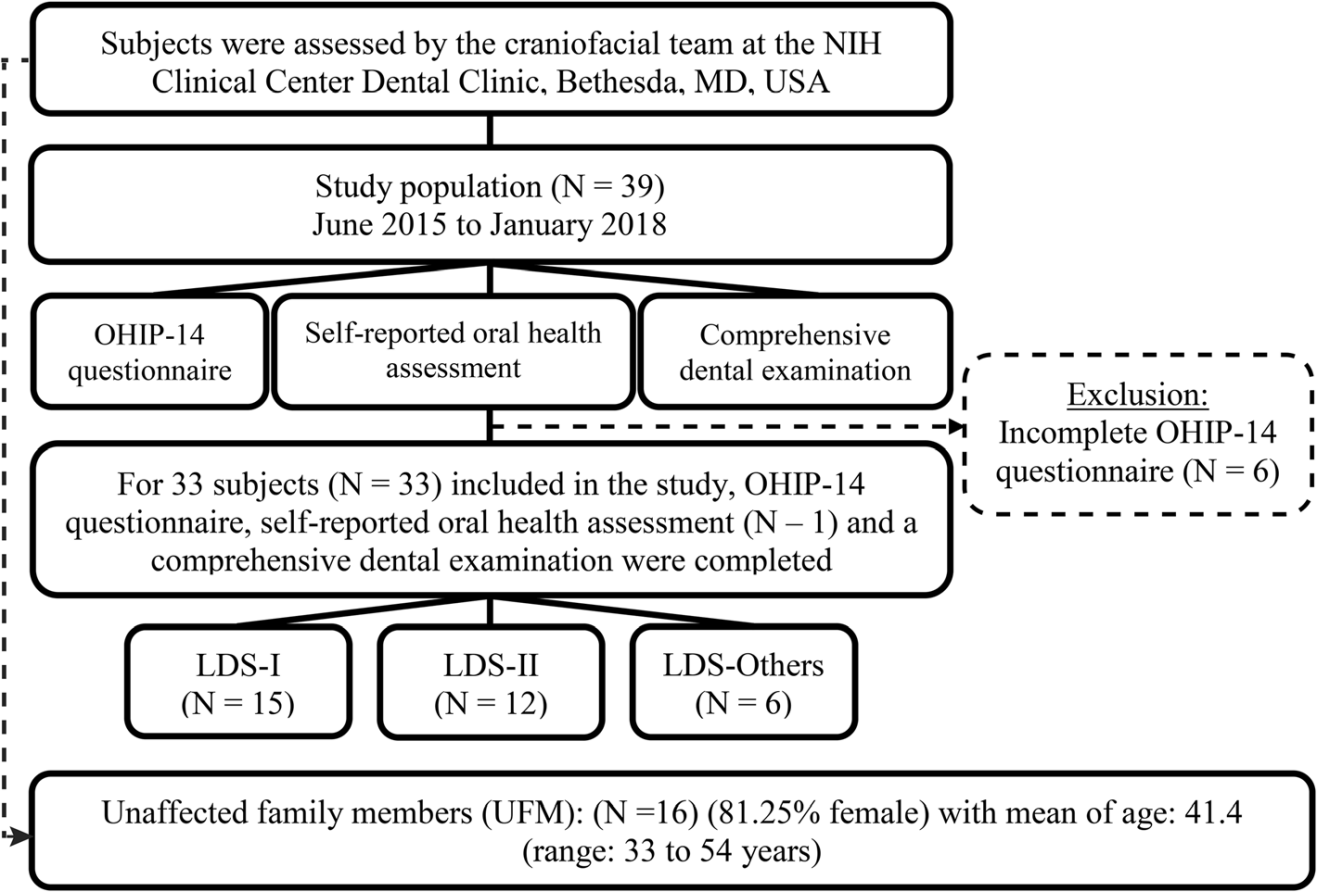
Study Population Flowchart. OHIP-14 = Oral Health Impact Profile – 14 questionnaire, LDS-I = TGF-β receptor 1 mutation (*TGFBR1*), LDS-II = TGF-β receptor 2 mutation (*TGFBR2*), LDS-Others = *SMAD3*, *TGFB2*, and *TGFB3* mutations combined

From June 2015 to January 2018, subjects who were genetically and clinically diagnosed with Loeys-Dietz syndrome were referred to the National Institute of Allergy and Infectious Diseases as part of the NIH IRB-approved *Natural History and Genetics of Food Allergy and Related Conditions study* (#15-I-0162). Subsequently, LDS subjects were evaluated at the National Institutes of Health Clinical Center (NIH CC) Dental Clinic as part of the NIH IRB-approved *Natural History of Craniofacial Anomalies and Developmental Growth Variants study* (#16-D-0040); consent was obtained from all enrollees. For each subject, the OHIP-14 questionnaire (Additional file 1: Table S1), a self-reported oral health assessment (a non-validated 25-question health survey formulated by the authors) and a comprehensive dental examination were obtained as part of the natural history study. To be included, subjects had to be ≥2 years old with a confirmed diagnosis of LDS and had completed the OHIP-14 questionnaire. For subjects who were ≤ 8 years old (*N* = 9), the guardian had completed the OHIP-14 questionnaire on their behalf. Of the 39 subjects with LDS seen at the NIH CC during the study period, 33 met inclusion criteria; 15 subjects had *TGFBR1* mutation (LDS-I), 12 subjects had *TGFBR2* mutation (LDS-II), and 6 subjects had either *SMAD3*, *TGFB2*, or *TGFB3* mutations (LDS-Others). Sixteen unaffected family members (UFMs) were also included in this study as controls (Fig. 1).

### Data collection

OHRQoL was measured using the OHIP-14 questionnaire that encapsulates seven health domains with respect to problems with the teeth, dentures or mouth [4, 5]:

1. Functional limitation – trouble with pronunciation and impaired taste.
2. Pain – aching in the mouth and discomfort when eating.
3. Discomfort – self-consciousness and tense feeling.
4. Physical disability – unsatisfactory diet and necessity to interrupt meals.
5. Psychological disability – difficulty relaxing and self-embarrassment.
6. Social disability – irritability with other people and difficulty doing usual jobs.
7. Handicap – less satisfaction in life and inability to function.

A score of 0–8 could be achieved in each health domain that is comprised of two questions. A standardized value was assigned for each answer on the following frequency scale: 0 = never, 1 = hardly ever, 2 = occasionally, 3 = often, and 4 = very often. A maximum score of 56 could be achieved with higher scores corresponding to worse OHRQoL.

To assess oral health self-care behavior (OHSB), 4 questions were extracted from a self-reported oral health assessment questionnaire consisting of 25 questions (non-validated) formulated by the authors. The 4 questions asked were:

1. Do you receive regular dental care?
  Response: yes (visits to the dentist every 6 to 12 months) or no.
2. What was the main reason for the last dental visit?
  Response: free response (responses were subsequently grouped as either routine maintenance or acute problem categories).
3. Overall, how would you rate your oral hygiene routine?
  Response: multiple choices of either poor, fair, good, very good, or excellent.
4. Overall, how would you rate the health of your teeth and gums?
  Response: multiple choices of either poor, fair, good, very good, or excellent.

**Fig. 2:**
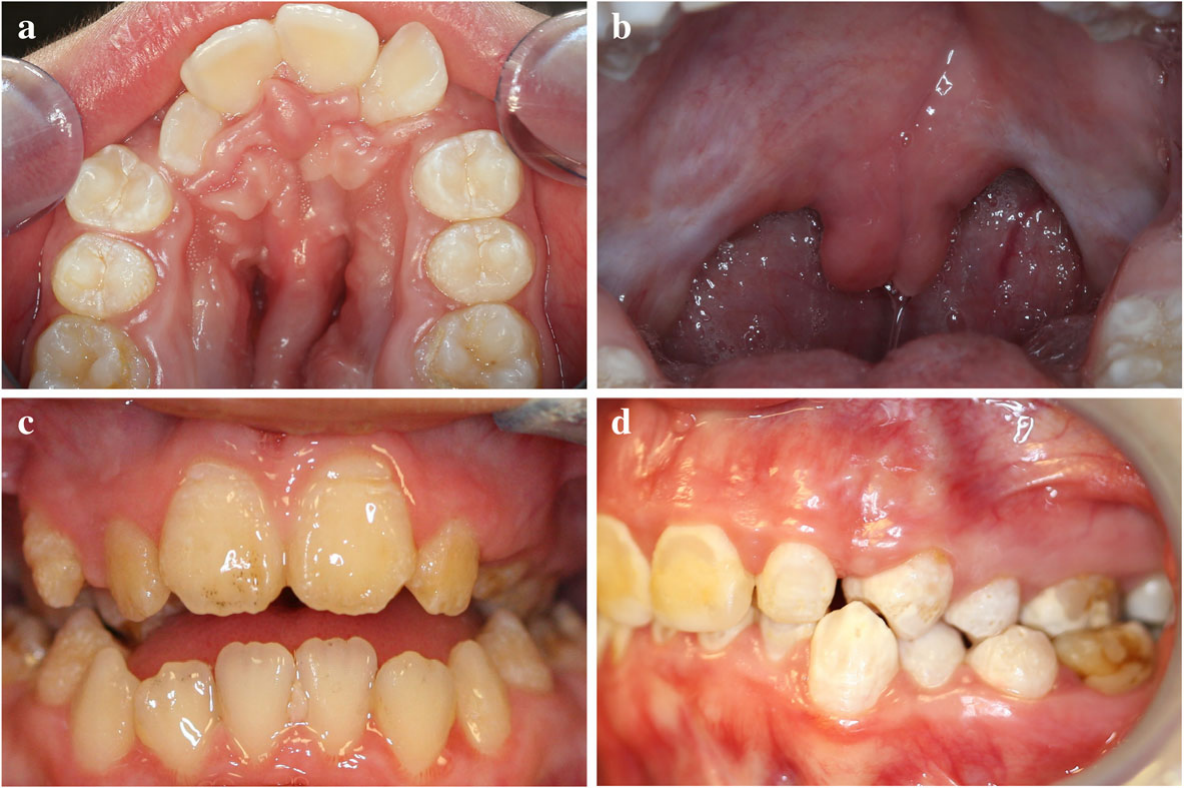
Representative Photos of Oral Manifestations in LDS Cohort. **a** High-arched and narrowed palate; **b** bifid uvula; **c** structural enamel defect includes pitting enamel and horizontal grooves on enamel surface; **d** malocclusion includes dental crowding and crossbite

Experienced dentists at the NIH CC Dental Clinic examined each patient and included details about the oral structures and soft tissue, dentition and periodontal health, dental occlusion, and temporomandibular joints (TMJ). Extra- and intra-oral photos and panoramic radiographs were taken to support the assessment. Similar features that were identified in the oral examination were grouped together to create a total of five abnormal oral manifestation categories (variables) for LDS (Fig. 2):

1. Abnormal soft and hard palate – high-arched, V-shaped, narrow palate, and/or bifid uvula (split/cleft of the uvula).
2. Gingivitis – signs of gum inflammation or active bleeding on probing.
3. Structural anomalies causing dental hypersensitivity – gingival recession, dentinal hypersensitivity to hot or cold temperature, and/or structural enamel defect. Intra-oral photos and radiographs were utilized to further evaluate enamel defects. Structural enamel defects were defined as rough, chipped, grooved, or pitted on the enamel surfaces. Subjects experienced one or more of these structural anomalies self-reported tooth hypersensitivity.
4. Malocclusion – dental crowding and abnormal inter-arch relationship. Dental crowding was evidenced by crowding of teeth or orthodontic treatment as a treatment of crowding. Abnormal inter-arch relationship was evidenced by overjet (> 3 mm), overbite (> 50%), anterior and/or posterior crossbite (bilateral and unilateral), and/or Angle’s Classification of Class II or Class III using first molars as primary reference and/or canines as secondary reference (if appropriate).
5. TMJ abnormalities - limited range of motion (< 30 mm), pain at rest or at function, and/or joint sounds with popping and clicking (bilateral and unilateral).

All data were entered and maintained in a secure database (REDCap) [20] by highly trained clinical study coordinators.

### Statistical analysis

The descriptive statistical analyses consisted of univariate analyses for LDS and unaffected family members (UFMs) subjects including mean, standard deviation, and range. Bivariate and multivariate analyses were carried out to identify categorical variables associated with the OHRQoL as represented by OHIP-14 scores (primary outcome) using the Wilcoxon Rank-Sum test. The relationships among self-rated daily oral hygiene and self-reported oral health status to OHIP-14 scores were derived using Pearson Correlation Coefficient. Unadjusted and parsimonious linear regression models were conducted to assess the relationship between each oral manifestation and OHIP-14 scores. Two additional variables included in the regression analyses were self-reported poor-to-fair oral health status and cumulation of four or more of the 5 oral manifestations listed above and the self-reported oral health status. All statistical analyses were performed using GraphPad Prism version 8.0.2 (263) for Windows (GraphPad Software, La Jolla, CA) and SAS version 9.4 (SAS Institute Inc., Cary, NC) with significance level of *p* ≤ 0.05.

## Results

### Patients with LDS have poor OHRQoL

A total of 33 subjects with LDS were included in the study; 51.5% were female and the mean age was 19.6 ± 15.1 years (range: 3–57 years). There were 16 UFMs who were included as controls; 81.3% were female and the mean age was 41.4 ± 6.1 years (range: 33–54 years; Table 1). In a bivariate analysis, the primary outcome or OHRQoL (represented by OHIP-14 score) was assessed by age, gender, type of mutation, and mode of inheritance for LDS subjects as potential confounders (Table 2). All findings were non-significant indicating that these independent variables do not have any influence on OHRQoL for LDS patients, even when looking at individual OHIP-14 subscale health domains (Additional file 1: Tables S2-S4).

**Table 1:** Subject Characteristics

Variables	LDS	UFM
Total subjects (n)	33	16
Gender		
Male, % (n)	48.5 (16)	18.75 (3)
Female, % (n)	51.5 (17)	81.25 (13)
Age		
Mean (Years)	19.6^#^	41.4
SD	15.1	6.11
Range	3–57	33–54

Statistical analyses: A paired sample t-test. ^#^Significant compared to controls (*p* < 0.001)

*LDS* Loeys-Dietz Syndrome

*UFM* Unaffected Family Member

**Table 2:** Comparison of OHIP-14 Scores by Age, Gender, Type of Mutation and Mode of Inheritance in LDS

Variables	% (n)	OHIP-14 Mean ± SD	*P*-value
Age			ns
Childhood (< 11 yrs)	30.3% (10)	4.80 ± 6.3	
Adolescence (> 11 to < 18 yrs)	36.4% (12)	5.17 ± 5.4	
Adulthood (> 18 yrs)	33.3% (11)	8.18 ± 7.6	
Gender			ns
Male	48.5% (16)	6.38 ± 6.6	
Female	51.5% (17)	5.76 ± 6.5	
Type of Mutation			ns
TGFBR1	45.4% (15)	6.13 ± 7.4	
TGFBR2	36.4% (12)	6.00 ± 7.4	
Others	18.2% (6)	6.00 ± 7.0	
Mode of Inheritance			ns
Sporadic	83.8% (26)	6.50 ± 6.4	
Familial	21.2% (7)	4.43 ± 6.9	

Statistical analysis: Wilcoxon Rank-Sum test was employed. *ns* not significant

Total OHIP-14 scores were significantly higher in LDS subjects (6.30 ± 6.37) when compared to UFMs (1.50 ± 2.28), *p* < 0.01 (Table 3), and higher than the previously reported scores of the general U.S. population (2.81 ± 0.12) [3]. Of note, most of the unaffected family members included in this study were adult females (81.3%), which may be seen as a limitation for the comparison with the affected group. However, based on the literature, OHIP-14 has been extensively reported to be gender-neutral for both the general population [4] and patients with other rare connective tissue disorders [8–11]. Moreover, as shown above, both gender and age were found to have no significant effect on OHIP-14 scores in the cohort of patients with LDS.

**Table 3:** Comparison of OHIP-14 Score between LDS and UFM

OHIP-14 Domains	LDS	UFM	*P*-value
	OHIP-14 Mean ± SD		
Functional Limitation	0.85 ± 1.3	0.22 ± 0.6	**0.05**
Pain	1.36 ± 1.5	0.41 ± 1.0	**< 0.05**
Discomfort	1.15 ± 1.6	0.68 ± 1.3	**0.05**
Physical Disability	0.88 ± 1.5	0.44 ± 0.9	**< 0.01**
Psychological Disability	1.00 ± 1.3	0.46 ± 1.1	**< 0.05**
Social Disability	0.52 ± 1.0	0.15 ± 0.4	ns
Handicap	0.30 ± 1.0	0.07 ± 0.3	ns
Total OHIP-14	6.30 ± 6.4	1.50 ± 2.3	**< 0.01**

Statistical analyses: Wilcoxon Rank-Sum test was employed. *ns* not significant. Bold values are significant (*p* ≤ 0.05)

*LDS* Loeys-Dietz Syndrome

*UFM* Unaffected Family Members

The OHIP-14 subscale health domains that contributed most significant to the difference between LDS subjects and UFMs were (*p* < 0.05): functional limitation (mean 0.85 vs 0.22), pain (mean 1.36 vs 0.41), discomfort (mean 1.15 vs 0.68), physical disability (mean 0.88 vs 0.44), and psychological disability (mean 1.00 vs 0.46). The social disability and handicap subscale health domains were not significant by multivariate analyses (Table 3).

### Oral health self-care behavior is not a major factor influencing OHRQoL in LDS patients

Of the 32 subjects who completed the 4 oral health self-care behavior (OHSB) questions, 26 subjects (81.3%) had access to regular dental care, 24 (75.0%) had scheduled routine care (i.e routine check-ups, orthodontic treatment, dental hygiene) as the most common reason for dental visits (75.0%), and 24 (75.0%) had self-rated good-to-excellent daily oral hygiene routine, and 21 (65.6%) had self-rated good-to-excellent oral health (Table 4). There were no statistically significant differences in the OHIP-14 scores between LDS subjects who do not receive regular dental care when compared to those who do (mean 8.00 vs 5.35), and between LDS subjects who seek dental assistance for problem-based acute care as opposed to routine maintenance (mean 8.63 vs 4.92). However, the OHIP-14 scores were generally lower, indicating better OHRQoL, for subjects with access to care and with routine maintenance. No correlation was found between self-rated daily oral hygiene routine and OHIP-14 scores. However, a strong correlation was observed between self-reported oral health status (excellent-to-poor) and OHIP-14 scores (*p* < 0.0001) (Table 4), which highlights the fact that OHIP-14 scores are consistent with self-perception of oral health status in the LDS cohort.

**Table 4:** Clinical Characteristics of Oral Health Self-Care Behavior in LDS

Oral Health Self-Care Behavior Questions	Total LDS (*N* = 32)		
	% subjects (n)	OHIP-14 Mean ± SD	*P*-Value
^a^Do you receive regular dental care?			ns
Yes	81.25 (26)	5.35 ± 5.34	
No	18.75 (6)	8.00 ± 8.10	
^a^Reason to visit the dentist			ns
Check-up, orthodontic treatment or cleaning	75.00 (24)	4.92 ± 4.90	
Acute problem (Infection, Extraction, Filling)	25.00 (8)	8.63 ± 7.93	
^b^Overall, how would you rate your hygiene routine (regular tooth brushing, flossing, and your home oral mouth rinse)?			ns
Poor	6.25 (2)	8.00 ± 9.90	
Fair	18.75 (6)	8.33 ± 5.28	
Good	37.5 (12)	5.92 ± 6.04	
Very Good	28.12 (9)	5.44 ± 6.09	
Excellent	9.38 (3)	0.33 ± 0.58	
^b^Overall, how would you rate the health of your teeth and gums? (Oral health status)			**< 0.0001**
Poor	15.63 (5)	13.20 ± 4.49	
Fair	18.75 (6)	8.83 ± 4.71	
Good	34.38 (11)	5.00 ± 5.59	
Very Good	25.00 (8)	1.63 ± 1.99	
Excellent	6.25 (2)	0 ± 0	

Statistical analyses: ^a^Wilcoxon Rank-Sum and ^b^Pearson Correlation Coefficient tests were employed. *ns* not significant. Bold values are significant (*p* ≤ 0.05)

### Specific oral manifestations correlate with poor OHRQoL in LDS patients

**Fig. 3:**
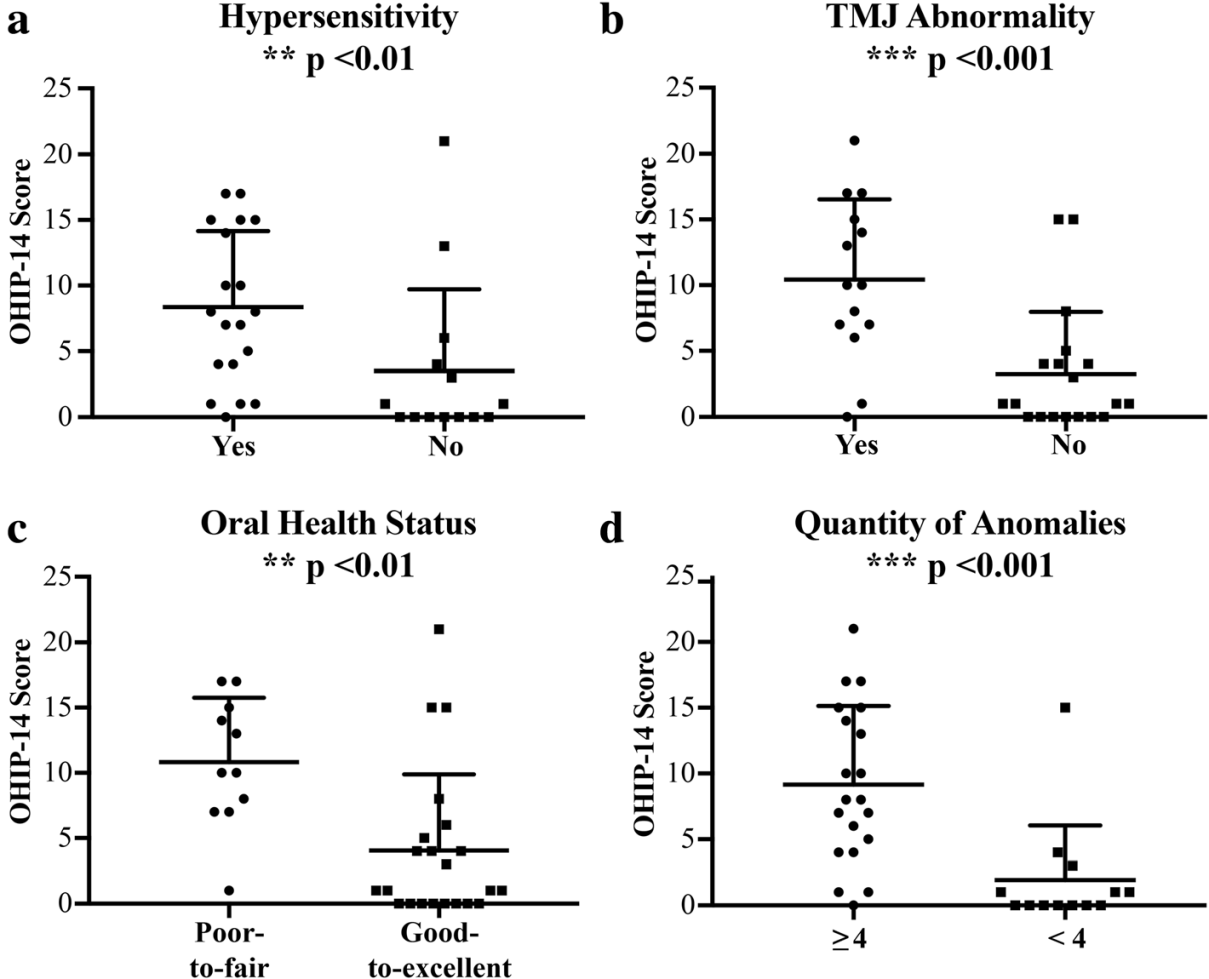
Dot plots of significant Oral Manifestations in LDS Cohort. Hypersensitivity (**a**), TMJ abnormalities (**b**), oral health status (**c**), and quantity of anomalies (**d**) significantly affect OHIP-14 score. Statistical analyses: Mann-Whitney U test was employed

Detailed characterization and illustration of the oral-dental anomalies found in the cohort of patients with LDS will be reported in a separate manuscript (unpublished data). The most common abnormal oral manifestations or variable in LDS subjects were (in descending order): malocclusion (97.0%), abnormal soft and hard palate (87.9%), gingivitis (60.6%), cumulation of four or more oral manifestations (60.6%), hypersensitivity (57.6%), TMJ abnormalities (42.4%), and poor-to-fair self-reported oral health status (33.3%). These oral manisfestations are seen in both primary and permanent dentitions. While malocclusion and hypersensitivity seem to equally affect children, adolescents and adults, abnormal hard and soft palate, gingivitis and TMJ abnormalities may get worse with age as they are more frequent in adolescents and adults than in children (Additional file 1: Table S5). The association between these oral manifestations and OHIP-14 scores were determined using regression models (Table 5). Unadjusted linear regression model showed a statistically significant association between OHIP-14 scores and four of the seven variables: hypersensitivity (β = 5.24; *p* < 0.05), temporomandibular joints (TMJ) abnormalities (β = 5.92; *p* < 0.01), self-reported poor-to-fair oral health status (β = 6.77; *p* < 0.01), and cumulation of four or more oral manifestations (β = 7.23; *p* < 0.001). Mann-Whitney U test was used to confirm the significant variables (Fig. 3). Finally, using a parsimonious model, self-reported poor-to-fair oral health status (β = 5.87, *p* < 0.01) and TMJ abnormalities (β = 4.95, *p* = 0.01) remained significantly associated with worse OHIP-14 scores.

**Table 5:** Crude Model of OHIP-14 Scores in Respect to Each Abnormal Variable in LDS

Oral manifestation feature	% subjects affected (n)	OHIP-14 Mean ± SD	β Coefficient	Standard Error	*P*-value
Five abnormal manifestations					
Malocclusion	97.0 (32)	6.41 ± 6.4	3.41	6.64	ns
Abnormal soft and hard palate	87.9 (29)	6.03 ± 6.3	−2.22	3.43	ns
Gingivitis	60.6 (20)	7.00 ± 6.3	1.77	2.28	ns
Hypersensitivity	57.6 (19)	8.53 ± 6.1	**5.24**	2.07	**< 0.05**
TMJ abnormality	42.4 (14)	9.71 ± 6.7	**5.92**	2.01	**< 0.01**
Two additional variables					
Self-reported poor-to-fair oral health status	33.3 (11)	10.82 ± 4.9	**6.77**	2.06	**< 0.01**
Cumulation of oral manifestations (≥ 4)	60.6 (20)	9.15 ± 6.0	**7.23**	1.90	**< 0.001**

Statistical analyses: Crude regression model was employed. *ns* not significant. Bold values are significant (*p* ≤ 0.05)

The patients in this report were initially enrolled as part of a study on food allergies, which may in some ways affect OHRQoL by influencing feeding behavior. Therefore, we tested the potential impact of food allergies on OHIP-14 score. In our cohort, OHIP-14 scores were not significantly different between patients who had food allergies and patients who did not (Additional file 1: Table S6).

Wilcoxon Rank-Sum test showed three of the seven OHIP-14 subscale health domains to be significantly associated with hypersensitivity: pain (*p* < 0.01), discomfort (*p* = 0.05), and psychological disability (*p* < 0.01). Wilcoxon Rank-Sum test also showed three of the seven OHIP-14 subscale health domains to be significantly associated with TMJ abnormalities: physical disability (*p* < 0.01), psychological disability (*p* < 0.05), and social disability (*p* < 0.05) (Table 6).

**Table 6:** Correlation Between Significant Abnormal Manifestations and OHIP-14 Domains

Dimension	Hypersensitivity			TMJ Abnormality		
	Yes	No	*P*- value	Yes	No	*P*- value
	OHIP-14 Mean ± SD			OHIP-14 Mean ± SD		
Functional Limitation	0.79 ± 1.1	0.93 ± 1.4	ns	1.14 ± 1.2	0.63 ± 1.3	ns
Pain	2.11 ± 1.5	0.50 ± 0.9	**< 0.01**	2.00 ± 1.7	1.00 ± 1.3	ns
Discomfort	1.79 ± 1.8	0.64 ± 1.0	**0.05**	1.79 ± 1.5	0.95 ± 1.6	ns
Physical Disability	1.26 ± 1.8	0.43 ± 1.1	ns	1.71 ± 1.9	0.32 ± 0.9	**< 0.01**
Psychological Disability	1.47 ± 1.5	0.36 ± 0.7	**< 0.01**	1.50 ± 1.3	0.63 ± 1.2	**< 0.05**
Social Disability	0.68 ± 1.1	0.29 ± 0.7	ns	0.93 ± 1.2	0.21 ± 0.6	**< 0.05**
Handicap	0.42 ± 1.2	0.14 ± 0.5	ns	0.64 ± 1.4	0.05 ± 0.2	ns

Statistical analyses: Wilcoxon Rank-Sum test was employed. *ns* not significant. Bold values are significant (*p* ≤ 0.05)

## Discussion

Rare diseases have a negative impact on general health-related quality of life for patients [21]. Several studies suggest that rare diseases can also have an undesirable impact on the oral health-related quality of life due to associated abnormal dental and craniofacial findings [8–11, 22–24]. Our study demonstrates for the first time that patients with LDS have worse OHRQoL when compared to both unaffected family members and the general U.S. population [4].

The OHIP-14 questionnaire is one of the most commonly used and validated surveys for determining OHRQoL; however, the survey does not specifically ask which oral manifestation is associated with OHRQoL health domains in cases where there are multiple anomalies. In the case of patients with LDS who have a wide-spectrum of dental, oral, and craniofacial anomalies, functional limitation, pain, discomfort, physical disability and psychological disability were greatly affected among the seven OHIP-14 subscale health domains. Here, we report that pain, discomfort and psychological disability are significantly correlated with dental hypersensitivity. In the clinical exams of patients with LDS, significant and unusual structural enamel or dentinal defects were noted in the clinical records that involved mild defects of enamel pitting or grooves to severe defects of absent enamel on the contact surfaces of teeth (unpublished data) that may contribute to the hypersensitivity and pain described by the patients. Such defects can affect the appearance of the teeth leading to self-consciousness (discomfort) and self-embarrassment (psychological disability) for patients with LDS.

A previous study has shown that temporomandibular disorders (TMD) have a negative impact on OHRQoL with discomfort and psychological disability being the most often affected OHIP-14 subscales for TMD patients [25]. Similarly, we also found a strong correlation of physical, psychological and social disability with TMJ abnormalities.

In the general population, inability to access to dental care was found to have the greatest impact on worse OHRQoL [3]. For patients with LDS, despite the rare condition, 81% reported receiving regular dental care which is much greater than the general population of children (59%) and adolescents (48%) [26]. Additionally, 75% of patients with LDS reported maintaining good-to-excellent daily oral hygiene. This strongly suggests that worse OHRQoL and higher OHIP-14 scores in patients with LDS was likely due to the underlying disease and the compilation of oral manifestations and symptoms as a result of the syndrome rather than oral health neglect. The patients with LDS who had limited access to dental care and received problem-based care (assumed to be episodic care and not part of routine or maintenance) did, in fact, have worse OHRQoL or higher OHIP-14 scores than those who had access to dental care, but this difference was not statistically significant. Based on our findings and given the presence of unusual oral manifestations, patients with LDS require appropriate dental interventions to ensure optimal OHRQoL beyond the scope of routine oral hygiene. The appropriate dental interventions include composite restorations, crowns and veneers to address the enamel defects and gingival recession that may be contributing to the hypersensitivity.

Knowledge of the oral manifestations that impact OHRQoL can provide valuable information to clinicians and assist in treatment planning and prioritizing care. We report that dental hypersensitivity, TMJ abnormalities, and a cumulation of four or more oral anomalies worsen OHRQoL for patients with LDS. Specific abnormalities such as enamel loss with exposure of dentin, enamel irregularity may pinpoint to an underlying dentin-enamel developmental problem due to the LDS-causing mutations. And the TMJ limitation of movement and pain may due to the collagen defect as a result of LDS-causing mutations. While malocclusion was not associated with worse OHRQoL, it is interesting to note that it was the most common oral manifestations and retrognathia was found in the majority of the LDS patients. This malocclusion and dentofacial deformity may contribute to TMJ abnormalities as retrognathia is often associated with TMJ pain [27, 28]. Additionally, self-reported poor-to-fair oral health status correlated strongly with worse OHRQoL and supports the important role of good oral health to quality of life.

Additional studies on OHRQoL should to be conducted on pre- and post-dental interventions to further evaluate the impact of dental treatments on LDS patients. Moreover, understanding the characteristics of the enamel and dentin abnormalities in patients with LDS at the ultrastructural and biomechanical levels will be essential to define the most appropriate dental interventions. We anticipate that dental treatments such as direct and/or indirect restoration will greatly improve OHRQoL as the restorations will address the dental hypersensitivity as well as improve dental esthetics which may be a psychological burden on patients with LDS.

One of the limitations of this study and most studies of rare diseases is the reduced sample size, which impacts the power in the statistical analyses. Additionally, one of the common limitations of child OHIP-14 is using parents as proxy. Though some parents may have limited knowledge concerning their children’s OHRQoL, it is reported that parent and child reports show statistically significant correlation [29, 30]. It is recommended for future studies to obtain both parental and child OHIP-14 reports in order to fully represent the child OHRQoL. However, because of the unique resources of the NIH CC Dental Clinic, enrollment of one of the largest cohorts of this condition in a short period of time was possible, as well as a consistent and comprehensive oral examination of subjects with LDS and unaffected family members. Including family members without LDS as a critical control group is a strength of this study on a rare condition, as family members are exposed to the same environment, diet, and access to care. This study addresses a gap in knowledge of a rare disease and provides insight to the OHRQoL status, the oral health self-care behavior and the impact of oral manifestations on OHRQoL for patients with LDS.

## Conclusions

Patients with LDS had significantly worse OHRQoL (higher OHIP-14 scores) than unaffected family members and the general American population despite the majority of patients reporting access to regular dental care. The oral manifestations or factors that were associated with the worse OHRQoL included dental hypersensitivity, TMJ abnormalities, and a cumulation of four or more oral manifestations. The worse OHRQoL is potentially related to distinct dental, oral, and craniofacial anomalies associated with LDS and not dental neglect or limited dental care. Specific treatment guidelines are necessary to ensure optimal quality of life in patients diagnosed with LDS, with a particular focus on dental hypersensitivity and TMJ abnormalities.

## Supplementary information

**Additional file 1: Table S1.** Distribution of OHIP-14 questionnaire. **Table S2.** Comparison of OHIP-14 health subdomains between three different age groups in the LDS cohort. **Table S3.** Comparison of OHIP-14 health subdomains between males and females in LDS cohort. **Table S4.** Comparison of OHIP-14 health subdomains between types of LDS mutations. **Table S5.** Frequency of oral manifestations for each age group in the LDS cohort. **Table S6.** Influence of Food Allergy on OHIP-14 (OHRQoL) in LDS Patients.

## Data Availability

The datasets used and/or analyzed during the current study are available from the corresponding author (JSL) on reasonable request.

## Abbreviations

LDS: Loeys-Dietz syndrome
NIDCR: National Institute of Dental and Craniofacial Research
NIH/CC: National Institutes of Health Clinical Center
OHIP-14: Oral Health Impact Profile-14 Questionnaire
OHRQoL: Oral Health-Related Quality of Life
OHSB: Oral health Self Care Behaviour
TMJ: Temporomandibular Joint
TMD: Temporomandibular Disorder
UFM: Unaffected Family Member
