# Supplementary material for: Oral health-related quality of life in Loeys-Dietz syndrome, a rare connective tissue disorder: an observational cohort study
Source: Orphanet J Rare Dis. 2019 Dec 16;14:291. doi: 10.1186/s13023-019-1250-y (PMC6915860; doi:10.1186/s13023-019-1250-y)
Supplement: Supplementary file 1 — Additional file 1: Table S1. Distribution of OHIP-14 questionnaire. Table S2. Comparison of OHIP-14 health subdomains between three different age groups in the LDS cohort. Table S3. Comparison of OHIP-14 health subdomains between males and females in LDS cohort. Table S4. Comparison of OHIP-14 health subdomains between types of LDS mutations. Table S5. Frequency of oral manifestations for each age group in the LDS cohort. Table S6. Influence of Food Allergy on OHIP-14 (OHRQoL) in LDS Patients. [file 13023_2019_1250_MOESM1_ESM.pdf]

Additional file 1

Table S1. Distribution of OHIP-14 questionnaire

OHIP domains	Descriptive of each question	Total LDS (N = 33)<br>% subject yes (N)
Functional Limitation	OHIP 1 - Had trouble pronouncing words<br>OHIP 2 - Felt sense of taste has worsened	36.36 (12)
Pain	OHIP 3 - Had painful aching in the mouth<br>OHIP 4 - Found it uncomfortable to eat any foods	57.58 (19)
Discomfort	OHIP 5 - Have been self-conscious<br>OHIP 6 - Felt tense	51.52 (17)
Physical Disability	OHIP 7 - Had an unsatisfactory diet<br>OHIP 8 - Had to interrupt meals	30.30 (10)
Psychological Disability	OHIP 9 - Found it difficult to relax<br>OHIP 10 - Have been a bit embarrassed	42.42 (14)
Social Disability	OHIP 11 - Have been irritable with other people<br>OHIP 12 - Had difficulty doing usual jobs	21.21 (7)
Handicap	OHIP 13 - Felt life in general was less satisfying<br>OHIP 14 - Have been totally unable to function	12.12 (4)

Table S2: Comparison of OHIP-14 health subdomains between three different age groups in the LDS cohort

Dimension	Age			*P*- value
	Childhood	Adolescence	Adult	
	OHIP-14 Mean ± SD			
Functional Limitation	1.56 ± 1.6	0.58 ± 1.2	0.58 ± 0.9	0.14
Pain	1.22 ± 1.5	1.75 ± 1.5	1.25 ± 1.6	0.66
Discomfort	0.33 ± 0.7	1.75 ± 1.8	1.58 ± 1.6	0.09
Physical Disability	0.77 ± 1.6	0.42 ± 0.9	1.5 ± 1.9	0.23
Psychological Disability	0.67 ± 1.4	0.92 ± 1.1	1.17 ± 1.1	0.64
Social Disability	0.78 ± 0.9	0.33 ± 0.8	0.75 ± 1.2	0.57
Handicap	0.22 ± 0.7	0.08 ± 0.3	0.58 ± 1.4	0.43

Childhood: includes subjects whose age was 11 years old (y.o.) or less
Adolescence: includes subjects whose age was greater than 11 y.o. and less than 18 y.o.
Adult: includes subjects whose age was greater than 18 y.o.
Statistical analyses: ANOVA test was employed.

Table S3: Comparison of OHIP-14 health subdomains between males and females in LDS cohort

Dimension	Gender		*P*- value
	Female	Male	
	OHIP-14 Mean ± SD		
Functional Limitation	0.53 ± 1.0	1.18 ± 1.4	0.13
Pain	1.35 ± 1.6	1.50 ± 1.5	0.79
Discomfort	1.41 ± 1.5	1.12 ± 1.7	0.61
Physical Disability	1.00 ± 1.7	0.81 ± 1.5	0.73
Psychological Disability	0.88 ± 0.9	1.13 ± 1.7	0.61
Social Disability	0.59 ± 1.1	0.44 ± 0.8	0.66
Handicap	0.47 ± 1.3	0.13 ± 0.3	0.30

Statistical analyses: Mann-Whitney U test was employed.

Table S4: Comparison of OHIP-14 health subdomains between types of LDS mutations

Dimension	Type of LDS Mutations			*P*- value
	LDS1	LDS2	LDS3-5	
	OHIP-14 Mean ± SD			
Functional Limitation	1.13 ± 1.5	0.58 ± 1.0	0.67 ± 1.2	0.50
Pain	1.27 ± 1.4	1.42 ± 1.6	1.83 ± 1.8	0.75
Discomfort	1.40 ± 1.6	1.17 ± 1.7	2.00 ± 2.3	0.65
Physical Disability	0.67 ± 1.4	1.17 ± 1.9	1.00 ± 1.3	0.71
Psychological Disability	1.20 ± 1.6	0.83 ± 1.1	0.83 ± 1.0	0.74
Social Disability	0.40 ± 0.8	0.83 ± 1.3	0.17 ± 0.4	0.33
Handicap	0.07 ± 0.3	0.67 ± 0.1.5	0.17 ± 0.4	0.25

LDS1: LDS patients with TGFBR1 mutation
LDS2: LDS patients with TGFBR2 mutation
LDS3-5: LDS patients with either SMAD3, TGFB2, or TGFB3 mutation
Statistical analyses: ANOVA test was employed.

Table S5: Frequency of oral manifestations for each age group in the LDS cohort

Oral manifestations	Age		
	Childhood (N = 10)	Adolescence (N = 12)	Adult (N = 11)
	% (N)		
Malocclusion	90.0% (9)	100.0% (12)	100.0% (11)
Abnormal soft and hard palate	40.0% (4)	50.0% (6)	81.8% (9)
Gingivitis	30.0% (3)	83.3% (10)	63.6% (7)
Hypersensitivity	90.0% (9)	100.0% (12)	72.7% (8)
TMJ abnormality	20.0% (2)	33.3% (4)	72.7% (8)
Self-rated poor-to-fair oral health status	20.0% (2)	33.3% (4)	45.5% (5)
Cumulation of oral manifestations (≥ 4)	20.0% (2)	75.0% (9)	81.8% (9)

Table S6: Influence of food allergy on OHIP-14 (OHRQoL) in LDS patients

Variables		% (n)	OHIP-14	*P*-value
			Mean ± SD	
Food Allergy				0.68
	Yes	30.3 % (10)	5.60 ± 1.7	
	No	69.7% (23)	6.61 ± 1.4	

Statistical analyses: Mann-Whitney U test was employed.
